# Association of typical atrial flutter and cavotricuspid isthmus ablation on clinical recurrence after cryoballoon ablation for atrial fibrillation

**DOI:** 10.3389/fcvm.2023.1303635

**Published:** 2023-12-15

**Authors:** Joo Hee Jeong, Hyoung Seok Lee, Yun Young Choi, Yun Gi Kim, Jong-Il Choi, Young-Hoon Kim, Hong Euy Lim, Il-Young Oh, Myung-Jin Cha, So-Ryoung Lee, Ju Youn Kim, Chang Hee Kwon, Sung Ho Lee, Junbeom Park, Ki-Hun Kim, Pil-Sung Yang, Jun-Hyung Kim, Jaemin Shim

**Affiliations:** ^1^ Division of Cardiology, Department of Internal Medicine, Korea University College of Medicine and Korea University Anam Hospital, Seoul, Republic of Korea; ^2^ Division of Cardiology, Hallym University Sacred Heart Hospital, Hallym University College of Medicine, Anyang, Republic of Korea; ^3^ Cardiovascular Center, Seoul National University Bundang Hospital, Seoul National University College of Medicine, Seongnam, Republic of Korea; ^4^ Heart Institute, Asan Medical Center, University of Ulsan College of Medicine, Seoul, Republic of Korea; ^5^ Department of Internal Medicine, Seoul National University Hospital, Seoul National University College of Medicine, Seoul, Republic of Korea; ^6^ Department of Internal Medicine, Heart Vascular and Stroke Institute, Samsung Medical Center, Sungkyunkwan University School of Medicine, Seoul, Republic of Korea; ^7^ Division of Cardiology, Department of Internal Medicine, Konkuk University Medical Center, Konkuk University School of Medicine, Seoul, Republic of Korea; ^8^ Department of Internal Medicine, Kangbuk Samsung Hospital, Sungkyunkwan University School of Medicine, Seoul, Republic of Korea; ^9^ Department of Cardiology, School of Medicine, Ewha Womans University, Seoul, Republic of Korea; ^10^ Department of Internal Medicine, Haeundae Paik Hospital, Inje University College of Medicine, Busan, Republic of Korea; ^11^ Department of Cardiology, CHA Bundang Medical Center, CHA University, Seongnam, Republic of Korea; ^12^ Department of Internal Medicine, Chungnam National University Hospital, Chungnam National University College of Medicine, Daejeon, Republic of Korea

**Keywords:** cryoballoon ablation, cavotricuspid isthmus, atrial fibrillation, atrial flutter, radiofrequency ablation

## Abstract

Typical atrial flutter commonly occurs in patients with atrial fibrillation (AF). Limited information exists regarding the effects of concurrent atrial flutter on the long-term outcomes of rhythm control. This study investigated the association between concurrent typical atrial flutter and cavotricuspid isthmus (CTI) ablation and the recurrence of atrial arrhythmia. The data were obtained from a multicenter registry of cryoballoon ablation for AF (*n* = 2,689). Patients who were screened for typical atrial flutter were included in the analysis (*n* = 1,907). All the patients with typical atrial flutter underwent CTI ablation. The primary endpoint was the late recurrence of atrial arrhythmia, including AF, atrial flutter, and atrial tachycardia. Among the 1,907 patients, typical atrial flutter was detected in 493 patients (25.9%). Patients with concurrent atrial flutter had a lower incidence of persistent AF and a smaller size of the left atrium. Patients with atrial flutter had a significantly lower recurrence rate of atrial arrhythmia (19.7% vs. 29.9%, *p* < 0.001). In patients with atrial flutter, the recurrence rate of atrial tachycardia or atrial flutter was more frequent (7.3% vs. 4.7%, *p* = 0.028), but the recurrence rate of AF was significantly lower (17.0% vs. 29.4%, *p* < 0.001). Atrial flutter has been identified as an independent predictor of the primary endpoint (adjusted hazard ratio, 0.704; 95% confidence interval, 0.548–0.906; *p* = 0.006). Typical atrial flutter in patients with AF may serve as a positive marker of the recurrence of atrial arrhythmia, and performing CTI ablation in this population is associated with a reduced likelihood of AF recurrence. Performing routine screening and ablation procedures for coexisting atrial flutter may improve the clinical outcomes of AF.

## 1. Introduction

Catheter ablation is the standard treatment strategy for symptomatic drug-refractory paroxysmal atrial fibrillation (AF). Pulmonary vein isolation (PVI) is most commonly achieved using radiofrequency catheter ablation (RFCA) (1). Cryoballoon ablation is an established technique for PVI in paroxysmal AF and is associated with superior outcomes for AF recurrence compared with antiarrhythmic drugs and non-inferior outcomes compared with RFCA (2, 3).

Typical atrial flutter is the most common form of macro-reentry atrial flutter and involves the cavotricuspid isthmus (CTI) (1). Typical atrial flutter commonly coexists with AF and is known to share a pathological process. Although previous studies have reported that PVI can significantly reduce the clinical recurrence of atrial arrhythmia in patients with AF and concurrent atrial flutter, subsequent studies have revealed that additional CTI ablation improves clinical outcomes by significantly reducing the recurrence of atrial flutter (4, 5). Therefore, linear ablation of the CTI is indicated in patients with concurrent atrial flutter (1). However, the association of concurrent typical atrial flutter and ablation with AF recurrence has not been established. In addition, although cryoballoon ablation is widely used as an initial ablation strategy for AF, it is not suitable for the ablation of typical atrial flutter. Consequently, CTI ablation using radiofrequency energy during cryoablation requires an additional catheter and system, resulting in a significant increase in costs and procedure duration (6). Accordingly, the typical clinical atrial flutter may be neglected in patients undergoing cryoballoon ablation for AF. Therefore, based on a multicenter cryoballoon ablation registry, we investigated the association between concurrent typical atrial flutter and CTI ablation and the recurrence of atrial arrhythmia.

## 2. Materials and methods

### 2.1. Study population

The data were obtained from a multicenter, retrospective, observational cohort of cryoballoon ablation in South Korea. Twelve tertiary centers capable of cryoballoon ablation for AF were included in the registry. Cryoballoon ablation was eligible for patients who had been diagnosed with AF based on 12-lead electrocardiography (ECG) and met the following criteria: (i) documented drug-refractory AF after a minimum of 6 weeks of treatment with standard-dose antiarrhythmic drugs or (ii) were unable to continue medication due to side effects, sick sinus syndrome, or bradycardia. Typical atrial flutter was diagnosed if the ECG resembled a counterclockwise atrial flutter (negative flutter wave at the inferior leads and positive flutter wave at v1) or clockwise atrial flutter (positive flutter wave at the inferior leads and negative flutter wave at v1). In cases where the flutter wave was not clear on the clinical ECG (i.e., Holter ECG), an attempt was made to induce typical atrial flutter with repeated rapid atrial pacing until a cycle length of 200 ms was achieved. Patients who underwent cryoballoon ablation for drug-refractory AF were included in the registry (*n* = 2,689). Patients who were screened for the presence of typical atrial flutter using (i) clinical ECG (12-lead ECG, or Holter ECG) and (ii) induced atrial flutter during the electrophysiologic study were included for further analysis (*n* = 1,907). This study complied with the principles of the Declaration of Helsinki and was approved by the Institutional Review Board of each hospital.

### 2.2. Procedure for cryoballoon and radiofrequency ablation

A transseptal puncture was performed to gain access to the left atrium, and a 15-Fr deflectable sheath (FlexCath, Medtronic) was advanced through the left atrium. A circular mapping catheter (Achieve; Medtronic) was used to record pulmonary vein potentials. Cryoenergy was delivered using a 28- or 23-mm balloon (second-generation Arctic Front Advance, Medtronic). The cryoballoon ablation procedure generally conformed to the practice guidelines for cryoballoon ablation in AF, and detailed approaches and dosing regimens, such as cryoenergy delivery time, fluoroscopic or intracardiac echocardiographic guidance for balloon positioning and occlusion, use of general anesthesia, and post-ablation testing, were applied at the operator's discretion (7).

After PVI, CTI ablation was performed in all patients with a typical atrial flutter. CTI ablation was not indicated in patients without documented or induced typical atrial flutter. When indicated, a linear lesion was created along the CTI image using a radiofrequency catheter under fluoroscopic guidance. The radiofrequency energy was set between 25 to 40 W during ablation, and an open-irrigated catheter was used. The choice of ablation catheter and settings were based on the operator's discretion. The CTI ablation was considered successful when a bidirectional block was demonstrated using differential pacing.

### 2.3. Follow-up and outcome measurement

The data on AF-related clinical variables were obtained, including demographic factors, medical history, clinical characteristics, and procedure-related variables. Complications were recorded during and after the procedures. Patients visited the clinic within 1–2 weeks after discharge and underwent regular follow-up appointments every 3–6 months, during which a 12-lead ECG was performed. A routine screening for AF recurrence with ambulatory ECG monitoring (Holter or patch ECG monitoring) was performed according to the guideline recommendations, and the time interval and type of ambulatory ECG monitoring were chosen according to the physician's discretion (1). If there was no evidence of recurrence following the cryoballoon ablation procedure, the antiarrhythmic medication was withdrawn during the follow-up period, and the timing of drug withdrawal was determined at the physician's discretion. The primary endpoint was defined as the late recurrence of atrial arrhythmia, including AF, atrial flutter, and atrial tachycardia, 90 days after the blanking period. The secondary endpoints included the following: (1) early recurrence of atrial arrhythmia during the blanking period, (2) late recurrence of AF, (3) late recurrence of atrial flutter or atrial tachycardia, (4) acute complications, and (5) major adverse cardiovascular events related to the procedure.

### 2.4. Statistical analysis

The categorical variables are described as numbers and percentages, and continuous variables are described as means and standard deviations. Student's *t*-test, Mann–Whitney U, chi-square, or Fisher's exact tests were used to compare variables, as indicated. Kaplan–Meier analysis and log-rank tests were used to assess time-dependent variables. Univariable and multivariable Cox proportional hazards models were used to adjust for covariates. To reduce selection bias, propensity score (PS) matching analysis was conducted to compare patients with and without atrial flutter. For PS matching analysis, the likelihood of atrial flutter was quantified using a multivariable logistic regression model. All previously specified baseline characteristics were included in the model (age, sex, body mass index, creatinine level, duration of AF, persistent AF, heart failure, hypertension, diabetes mellitus, previous stroke or transient ischemic attack, previous myocardial infarction, coronary artery disease, peripheral artery disease, chronic kidney disease, mitral valve disease, sick sinus syndrome, hypertrophic cardiomyopathy, implantable cardioverter defibrillator implant, pacemaker implant, CHA_2_DS_2_-VASc score, left atrial diameter, and left ventricular ejection fraction). After calculating the expected probabilities, we matched each patient in the atrial flutter group with those without atrial flutter at a 1:1 ratio using the nearest neighbor method with a caliper width of 0.2 of the standard deviation of the logit PS. PS matching resulted in 370 patients in the atrial flutter group and in those without atrial flutter. All variables used for PS matching were well-balanced (Supplementary Material Figure S1). All statistical analyses and model development were performed using the SPSS software (version 26; SPSS Inc., Chicago, IL, USA) and R Statistical software (version 4.2.3; R Foundation for Statistical Computing, Vienna, Austria).

## 3. Results

**Figure 1:**
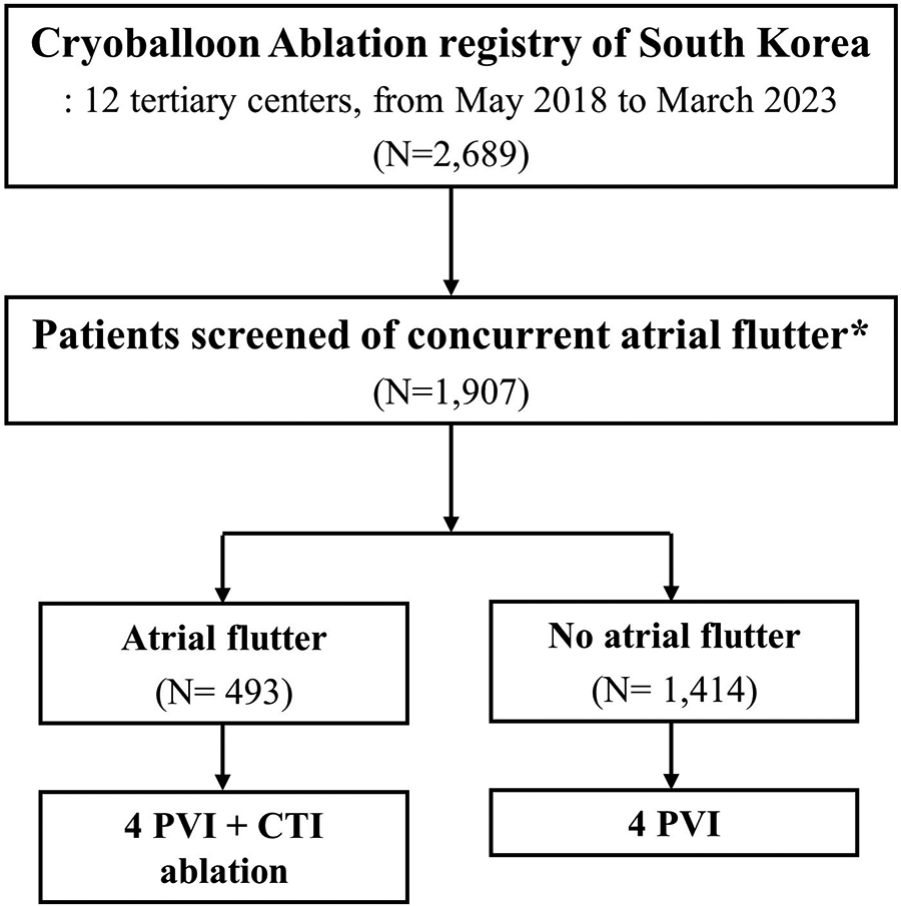
Flowsheet of the study. PVI, pulmonary vein isolation; CTI, cavotricuspid isthmus. *Concurrent atrial flutter was screened using (i) documented typical atrial flutter (on 12-lead ECG or Holter ECG) or (ii) induced typical atrial flutter during the electrophysiology study.

A total of 2,689 patients underwent cryoballoon ablation and were followed up between May 2018 and March 2023 (Figure 1). Consequently, 1,907 patients who were examined for concurrent atrial flutter using clinical ECG and electrophysiological studies were included in the analysis. Among the 1,907 patients, 493 (25.9%) patients were detected with typical atrial flutter and underwent CTI ablation in addition to PVI.

The baseline characteristics of the study cohort are presented in Table 1. In the total cohort, the mean age at the time of the procedure was 61.8 ± 10.4 years, and 1,449 (76.0%) of the patients were male. The mean CHA_2_DS_2_-VASc score was 2.1 ± 1.6. The mean duration from the first detection of AF to cryoballoon ablation was 3.1 ± 3.1 years, and 1,024 (53.7%) patients had persistent AF. The mean left atrial diameter was 43.3 ± 6.9 mm, and the mean left ventricular ejection fraction was 58.9% ± 8.8%. Compared with patients without atrial flutter, those with atrial flutter had a lower body mass index and a higher prevalence of comorbidities, including heart failure, diabetes mellitus, and a history of ischemic stroke or transient ischemic attack. Consequently, the patients with atrial flutter had higher CHA_2_DS_2_-VASc scores. In contrast, the patients with atrial flutter had a lower proportion of persistent AF, lower left atrial diameter, and lower left ventricular ejection fraction. The proportion of patients with cardioverter defibrillator implants differed significantly between the two groups.

**Table 1:** Baseline characteristics.

	Total cohort				PS-matched cohort (1:1)		
	Total (*n* = 1,907)	No atrial flutter (*n* = 1,414)	Atrial flutter (*n* = 493)	*p*-value	No atrial flutter (*n* = 370)	Atrial flutter (*n* = 370)	*p*-value
Age (years)	61.8 ± 10.4	61.6 ± 10.1	62.3 ± 11.2	0.203	61.0 ± 10.4	62.2 ± 10.6	0.120
Sex (male)	1,449 (76.0)	1,069 (75.6)	380 (77.1)	0.548	296 (80.0)	288 (77.8)	0.528
Body mass index (kg/m^2^)	25.6 ± 3.4	25.7 ± 3.5	25.2 ± 3.2	0.003	25.4 ± 3.6	25.3 ± 3.1	0.798
Creatinine (mg/dl)	1.0 ± 0.7	1.0 ± 0.6	1.0 ± 0.7	0.126	1.0 ± 0.7	1.0 ± 0.4	0.774
Duration of AF (years)^a^	3.1 ± 3.1	3.1 ± 3.2	3.0 ± 3.0	0.288	2.6 ± 2.6	3.0 ± 3.0	0.123
Persistent AF	1,024 (53.7)	783 (55.4)	241 (48.9)	0.015	165 (44.6)	180 (48.6)	0.302
Comorbidity							
Heart failure^b^	446 (23.4)	299 (21.1)	147 (29.8)	<0.001	105 (28.4)	102 (27.6)	0.870
Hypertension	1,142 (59.9)	844 (59.7)	298 (60.4)	0.809	204 (55.1)	215 (58.1)	0.458
Diabetes mellitus	404 (21.2)	278 (19.7)	126 (25.6)	0.007	88 (23.8)	90 (24.3)	0.931
Previous stroke or TIA	233 (12.2)	159 (11.2)	74 (15.0)	0.034	50 (13.5)	51 (13.8)	1.000
Previous myocardial infarction	26 (1.4)	19 (1.3)	7 (1.4)	1.000	1 (0.3)	5 (1.4)	0.219
Coronary artery disease	147 (7.7)	102 (7.2)	45 (9.1)	0.203	30 (8.1)	32 (8.6)	0.894
Peripheral artery disease	11 (0.6)	7 (0.5)	4 (0.8)	0.650	2 (0.5)	2 (0.5)	1.000
Chronic kidney disease	168 (8.8)	117 (8.3)	51 (10.3)	0.192	27 (7.3)	32 (8.6)	0.587
End-stage renal disease	64 (3.4)	48 (3.4)	16 (3.2)	0.989	11 (3.0)	11 (3.0)	1.000
Mitral valve disease^c^	16 (0.8)	13 (0.9)	3 (0.6)	0.715	1 (0.3)	2 (0.5)	1.000
Sick sinus syndrome	85 (4.5)	55 (3.9)	30 (6.1)	0.056	21 (5.7)	22 (5.9)	1.000
Hypertrophic cardiomyopathy	45 (2.4)	30 (2.1)	15 (3.0)	0.323	13 (3.5)	12 (3.2)	1.000
ICD implant	20 (1.0)	7 (0.5)	13 (2.6)	<0.001	4 (1.1)	5 (1.4)	1.000
Pacemaker implant	71 (3.7)	46 (3.3)	25 (5.1)	0.090	13 (3.8)	17 (4.6)	0.714
CHA_2_DS_2_-VASc score	2.1 ± 1.6	2.1 ± 1.5	2.4 ± 1.6	<0.001	2.1 ± 1.5	2.3 ± 1.6	0.246
Echocardiography							
Left atrial volume index (ml/m^2^)	49.0 ± 18.4	49.3 ± 18.8	48.3 ± 17.1	0.328	46.2 ± 18.1	47.8 ± 17.4	0.235
Left atrial diameter (mm)	43.3 ± 6.9	43.6 ± 6.9	42.4 ± 6.6	0.003	42.3 ± 7.0	42.5 ± 6.6	0.656
Left ventricular ejection fraction (%)	58.9 ± 8.8	59.4 ± 8.2	57.6 ± 10.3	0.001	58.1 ± 9.2	57.5 ± 10.2	0.392
E/e’	9.4 ± 3.7	9.3 ± 3.5	9.6 ± 4.2	0.262	9.3 ± 3.4	9.6 ± 4.1	0.532

CTI, cavotricuspid isthmus; AF, atrial fibrillation; TIA, transient ischemic attack; ICD, implantable cardioverter defibrillator.

^a^ AF duration was defined as the time from the first detection of AF to the date of cryoballoon ablation.

^b^ Heart failure was defined as a clinical diagnosis of heart failure or objective signs of reduced or mildly reduced left ventricular ejection fraction (<50%).

^c^ Mitral valve disease was defined as a moderate or severe mitral stenosis and/or a history of mitral valve surgery.

### 3.1. Recurrence of atrial arrhythmia

**Figure 2:**
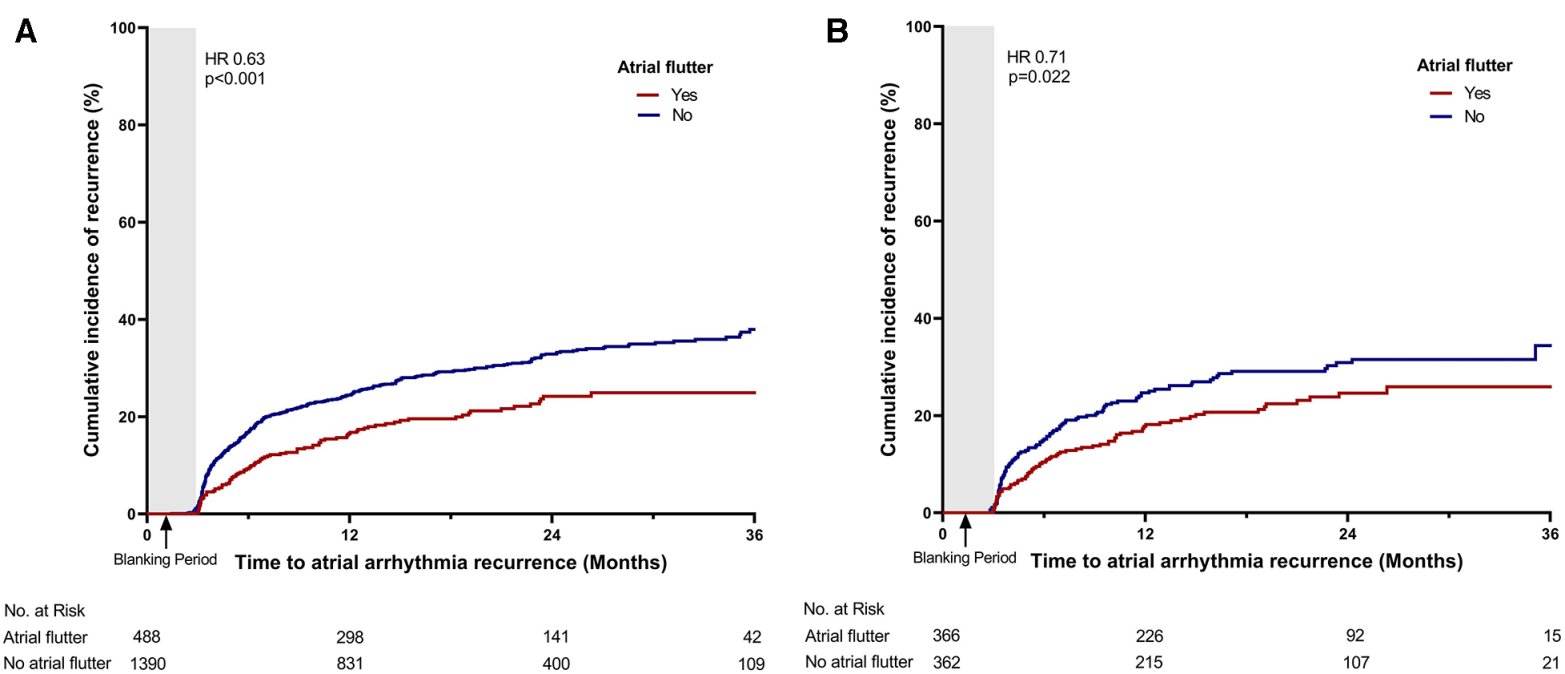
Time-to-event curve for the primary endpoint. Time-to-event curve for the primary endpoint (**A**) in the total cohort and (**B**) in the propensity score-matched cohort. HR, hazard ratio.

**Figure 3:**
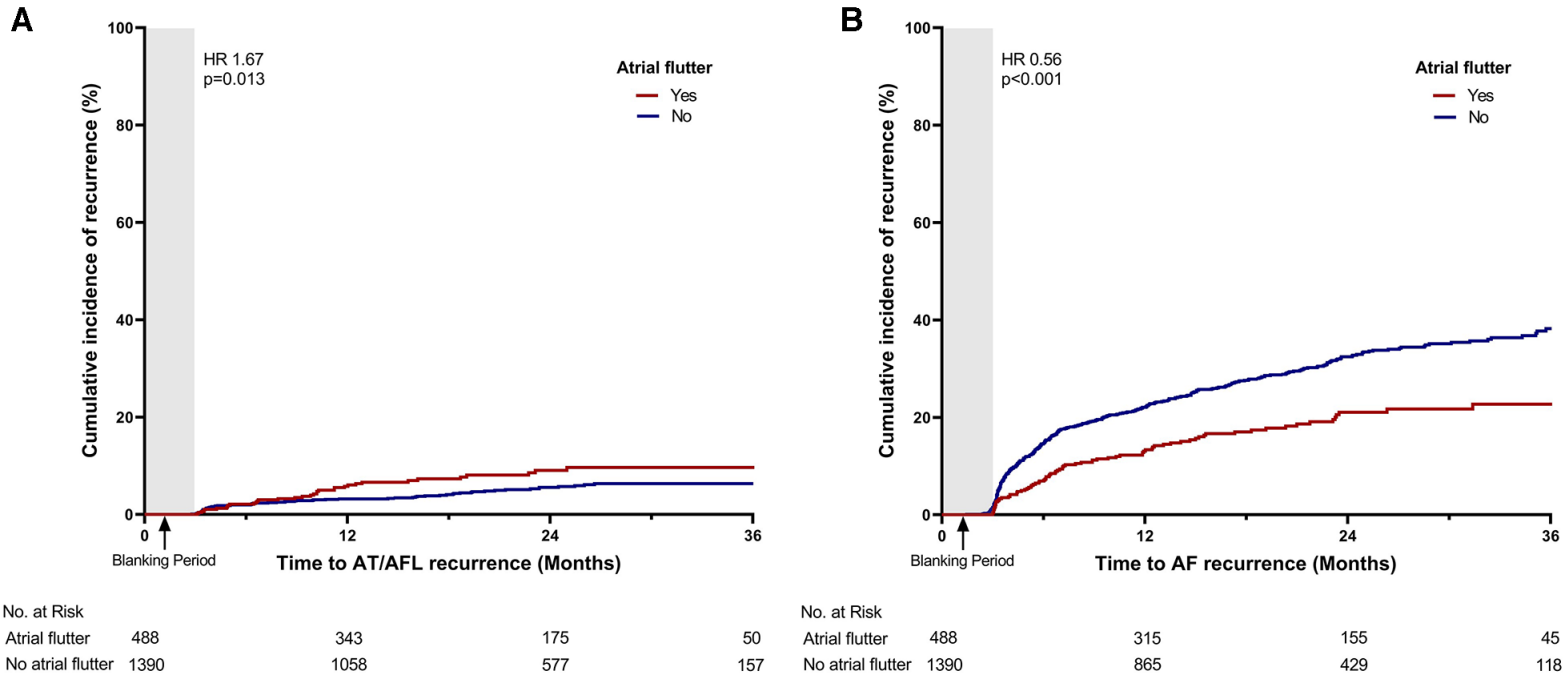
Time-to-event curve for the secondary endpoints. Time-to-event curve for the secondary endpoints: (**A**) late recurrence of atrial tachycardia or atrial flutter and (**B**) late recurrence of atrial fibrillation. HR, hazard ratio; AT, atrial tachycardia; AFL, atrial flutter; AF, atrial fibrillation.

The recurrence of atrial arrhythmia was assessed during the clinical follow-up. The mean duration of the clinical follow-up was 21.3 ± 11.9 months. A total of 29 patients were lost to follow-up during the control period. During the follow-up, 512 patients (27.3%) experienced recurrence of late atrial arrhythmia. Compared with patients without atrial flutter, those with atrial flutter presented a lower recurrence rate of atrial arrhythmia (19.7% vs. 29.9%, *p* < 0.001; Table 2, Figure 2A). Of the total cohort, 101 patients (5.4%) experienced recurrence of atrial tachycardia or atrial flutter. The patients with atrial flutter showed a higher recurrence rate of atrial tachycardia or atrial flutter (7.4% vs. 4.7%, *p* = 0.028; Table 2, Figure 3A). In contrast, late recurrence of AF was significantly lower in patients with atrial flutter (17.0% vs. 29.4%, *p* < 0.001; Table 2, Figure 3B).

**Table 2:** Primary and secondary endpoints.

	Total (*n* = 1,907)	No atrial flutter (*n* = 1,414)	Atrial flutter (*n* = 493)	*p*-value
Recurrence				
Early recurrence	500 (26.2)	401 (28.4)	99 (20.1)	<0.001
Late recurrence	512/1,878 (27.3)	416/1,390 (29.9)	96/488 (19.7)	<0.001
Atrial tachycardia or atrial flutter	101/1,878 (5.4)	65/1,390 (4.7)	36/488 (7.4)	0.028
AF	492/1,878 (26.2)	409/1,390 (29.4)	83/488 (17.0)	<0.001
Complication				
Any complication	103 (5.4)	73 (5.2)	30 (6.1)	0.506
Pericardial effusion	13 (0.7)	7 (0.5)	6 (1.2)	0.174
Pericardial effusion needing pericardiocentesis	5 (0.3)	2 (0.1)	3 (0.6)	0.217
Atrial-esophageal fistula	0 (0.0)	0 (0.0)	0 (0.0)	1.000
Access site complication	23 (1.2)	16 (1.1)	7 (1.4)	0.614
Access site complication needing intervention	4 (0.2)	4 (0.3)	0 (0.0)	0.045
Complete atrioventricular block	1 (0.1)	1 (0.1)	0 (0.0)	1.000
Transient phrenic nerve injury	48 (2.5)	34 (2.4)	14 (2.8)	0.716
Permanent phrenic nerve injury	0 (0.0)	0 (0.0)	0 (0.0)	1.000
Hemoptysis	10 (0.5)	7 (0.5)	3 (0.6)	1.000
Pulmonary vein stenosis	1 (0.1)	1 (0.1)	0 (0.0)	1.000
Gastroparesis	6 (0.3)	6 (0.4)	0 (0.0)	0.326
Major adverse cardiovascular event				
Cardiac surgery due to complication	1 (0.1)	0 (0.0)	1 (0.2)	0.581
Acute myocardial infarction	1 (0.1)	0 (0.0)	1 (0.2)	0.581
Cardiac arrest	0 (0.0)	0 (0.0)	0 (0.0)	1.000
Stroke	1 (0.1)	1 (0.1)	0 (0.0)	1.000
Death	1 (0.1)	0 (0.0)	1 (0.2)	0.581

AF, atrial fibrillation.

The univariable and multivariable Cox regression analysis revealed four independent predictors of the primary endpoint: duration of AF, persistent AF, left atrial diameter, and presence of atrial flutter (Table 3). Persistent AF was the strongest predictor of recurrence of atrial arrhythmia. Compared with paroxysmal AF, persistent AF resulted in 85% increased risk of atrial arrhythmia recurrence (*p* < 0.001). In addition, prolonged AF (per 1 year) and increased left atrial diameter (per 1 mm) each revealed a 4.0% and 5.1% increased risk of atrial arrhythmia recurrence, respectively. In addition to the baseline variables, the presence of atrial flutter resulted in a 39.6% decreased risk of atrial arrhythmia recurrence.

**Table 3:** Univariable and multivariable Cox regression analysis for primary endpoint.

	Unadjusted				Adjusted	
	Hazard ratio	95% confidence interval	*p*-value	Hazard ratio	95% confidence interval	*p*-value
Age (per 1 year)	0.994	0.986–1.002	0.166	0.983	0.967–0.998	0.028
Sex (male)	0.958	0.784–1.171	0.674	0.761	0.542–1.068	0.114
Body mass index (per 1 kg/m^2^)	1.017	0.992–1.043	0.195	0.966	0.937–0.996	0.026
Creatinine (per 1 mg/dl)	1.059	0.945–1.187	0.320	0.979	0.809–1.185	0.827
Duration of AF (per 1 year)	1.050	1.024–1.076	<0.001	1.040	1.012–1.069	0.005
Persistent AF	2.150	1.782–2.594	<0.001	1.850	1.489–2.313	<0.001
Atrial flutter	0.633	0.507–0.790	<0.001	0.704	0.548–0.906	0.006
Comorbidity						
Heart failure	1.049	0.858–1.282	0.641	0.823	0.580–1.167	0.274
Hypertension	0.879	0.737–1.047	0.148	0.851	0.629–1.152	0.296
Diabetes mellitus	0.956	0.779–1.196	0.748	0.970	0.693–1.359	0.860
Previous stroke or TIA	0.936	0.716–1.223	0.627	0.989	0.575–1.699	0.967
Previous myocardial infarction	0.649	0.269–1.566	0.336	0.734	0.297–1.815	0.503
Coronary artery disease	1.167	0.859–1.584	0.324	1.314	0.883–1.956	0.178
Peripheral artery disease	1.052	0.338–3.274	0.930	0.730	0.177–3.016	0.664
Chronic kidney disease	1.033	0.767–1.390	0.831	0.978	0.668–1.433	0.910
Mitral valve disease	1.609	0.763–3.393	0.211	0.415	0.129–1.334	0.140
Sick sinus syndrome	0.904	0.590–1.385	0.642	1.031	0.631–1.685	0.902
Hypertrophic cardiomyopathy	1.690	1.081–2.643	0.021	0.950	0.567–1.592	0.846
ICD implant	0.952	0.426–2.129	0.904	0.559	0.174–1.802	0.330
Pacemaker implant	0.944	0.582–1.531	0.815	1.132	0.640–2.004	0.670
CHA_2_DS_2_-VASc score (per 1 point)	0.969	0.917–1.024	0.261	0.959	0.763–1.205	0.718
Echocardiography						
Left atrial diameter (per 1 mm)	1.054	1.040–1.067	<0.001	1.051	1.034–1.067	<0.001
Left ventricular ejection fraction (per 1%)	0.993	0.984–1.002	0.129	0.997	0.985–1.010	0.671

AF, atrial fibrillation; TIA, transient ischemic attack; ICD, implantable cardioverter defibrillator.

PS matching was performed between the atrial flutter and non-atrial flutter groups to adjust for variables. After PS matching, no significant differences in baseline characteristics were found between the two groups (Table 1, Supplementary Material Table S1). In the PS-matched cohort, the atrial flutter group revealed a lower incidence rate of the primary endpoint (28.2% vs. 20.2%, *p* = 0.022, Figure 2B).

**Figure 4:**
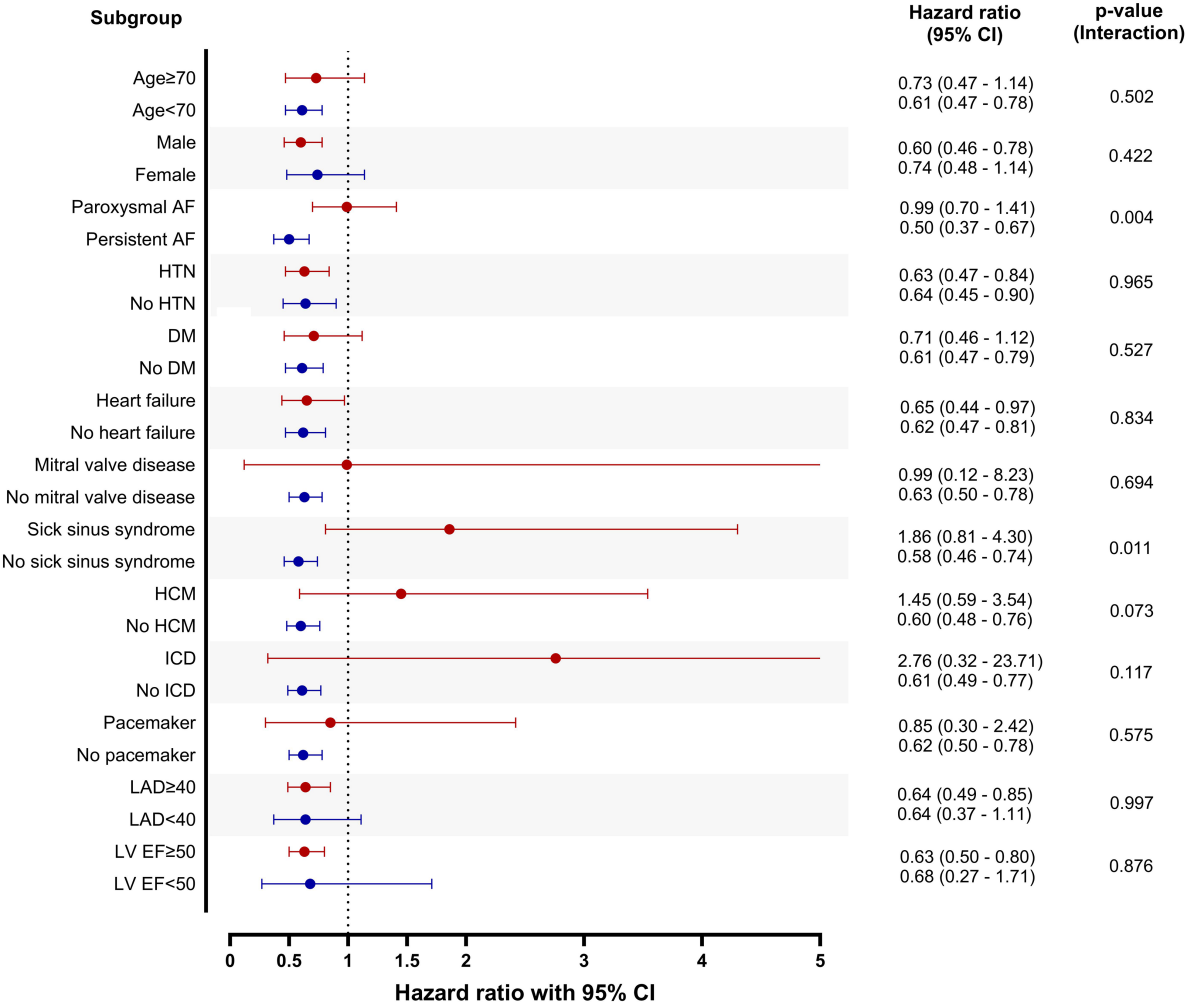
Subgroup analysis. Patients without atrial flutter were set as reference in each subgroup. CI, confidence interval; AF, atrial fibrillation; HTN, hypertension; DM, diabetes mellitus; HCM, hypertrophic cardiomyopathy; ICD, implantable cardioverter defibrillator; LAD, left atrial diameter; LV EF, left ventricular ejection fraction.

The risk of atrial arrhythmia recurrence was evaluated in various subgroups (Figure 4). The patients with persistent AF and those without sick sinus syndrome showed a stronger association between concurrent atrial flutter and recurrence of atrial arrhythmia.

### 3.2. Procedure-related complications

Acute and major adverse cardiac events were recorded post-procedure (Table 2). A total of 103 patients (5.4%) experienced acute complications after the procedure. Transient phrenic nerve injury was the most frequent (48 patients, 2.5%) and did not progress to permanent phrenic nerve injury in any patient. Access site complications were the second most frequent complication (23 patients, 1.2%), and four patients (0.2%) proceeded to intervention for such complications. Pericardial effusion occurred in 13 patients (0.7%), and five consecutive patients (0.3%) required pericardiocentesis. There were few major procedure-related adverse cardiovascular events: one patient experienced cardiac surgery due to complications (0.1%), one patient had acute myocardial infarction (0.1%), one patient suffered from stroke (0.1%), and one patient died (0.1%). The two groups had no significant difference in the prevalence of procedure-related acute complications or major adverse cardiovascular events.

## 4. Discussion

In the present study, we analyzed patients treated with cryoballoon ablation for drug-refractory AF and investigated the association of concurrent typical atrial flutter and CTI ablation on the long-term outcomes of rhythm control. Among patients with drug-refractory AF that received cryoballoon ablation, approximately one out of four (25.9%) exhibited concurrent typical atrial flutter and received additional CTI ablation. Patients with concurrent atrial flutter had a significantly lower recurrence of atrial arrhythmia after the blanking period. Although late recurrence of atrial tachycardia or atrial flutter was relatively frequent in patients with atrial flutter, AF recurrence was markedly lower, resulting in a lower incidence of overall atrial arrhythmia in patients with atrial flutter. The presence of typical atrial flutter was an independent predictor of atrial arrhythmia recurrence. Concurrent atrial flutter revealed a significantly stronger correlation with the recurrence risk in persistent AF than in paroxysmal AF. Our findings were validated using real-world data from a cryoballoon ablation registry. More than 1,900 patients from multiple tertiary centers were analyzed, and the outcome was robust, supporting active rhythm control in patients with concurrent atrial flutter.

### 4.1. Cryoballoon ablation in atrial fibrillation

Compared with previous landmark trials on cryoballoon ablation, the patients in our cohort were older and had a larger left atrium and higher CHA_2_DS_2_-VASc scores (2, 3). Although cryoballoon ablation is ideally recommended for paroxysmal AF with relatively less progressive atrial remodeling, real-world practice involves older patients with more advanced atrial remodeling and comorbidities. However, in our cohort, the atrial arrhythmia recurrence rate after cryoballoon ablation was lower than that reported in previous clinical trials (2, 3). This finding may be attributed to the absence of protocolized long-term ECG monitoring. This may also be explained by recent developments in ablative technologies and improved technical skills of operators, with various efforts to achieve optimal balloon positioning for durable lesions, such as the use of intracardiac echocardiography. Nonetheless, the beneficial effect of concurrent typical atrial flutter and CTI ablation is indisputable, which is associated with a decreased risk of atrial arrhythmia recurrence of up to 39% after adjusting confounding factors.

### 4.2. Atrial flutter and atrial fibrillation

CTI-dependent atrial flutter is the most common form of macro-reentry atrial flutter. The ablation procedure for CTI-dependent atrial flutter is relatively simple, with a high success rate and minimal risk of complications (8). However, atrial flutter commonly coexists with AF (5, 9). Patients with isolated typical atrial flutter without AF are highly likely to develop AF. A recent randomized trial that assessed CTI ablation vs. PVI using a cryoballoon in isolated typical atrial flutter revealed no significant difference in the recurrence of atrial flutter, whereas a significant reduction was found in the further occurrence of AF (10).

Conversely, more than one-third of patients with AF present with coexisting typical atrial flutter, necessitating CTI ablation in addition to PVI (9). An earlier randomized study comparing PVI alone with PVI and CTI ablation in patients with AF and typical atrial flutter revealed no significant difference in atrial arrhythmia recurrence, suggesting that PVI may be sufficient even in patients with concurrent atrial flutter (4). A similar study of 133 patients who underwent PVI for AF revealed that the recurrence of symptomatic atrial flutter was relatively common in patients with concurrent atrial flutter, which supported additional CTI ablation in AF patients with either spontaneous or induced typical atrial flutter (5). Consequently, CTI ablation and PVI are currently recommended in patients with AF and concurrent atrial flutter (1). However, prophylactic CTI ablation in patients without typical clinical atrial flutter is not recommended, as no clinical benefit has been found with additional CTI ablation (11, 12). To date, there is a lack of data regarding the effect of concurrent typical atrial flutter and its ablation on the likelihood of clinical recurrence of atrial arrhythmia, particularly in terms of recurrence as AF.

### 4.3. Mechanisms

A higher recurrence of atrial tachycardia seen in patients with typical atrial flutter is a natural consequence, as atrial flutter could recur if a conduction gap occurs in the linear lesions of the CTI. However, the reason for the lower AF recurrence in the concurrent atrial flutter group is not straightforward. This finding is supported by several mechanisms. First, patients with concurrent atrial flutter had relatively non-persistent AF and a smaller left atrium. A similar finding was reported in a previous prospective study that revealed a higher prevalence of paroxysmal AF and a smaller left atrium in patients with concurrent atrial flutter (11). A larger left atrium with progressive atrial remodeling is a structural substrate of AF, leading to a higher AF burden. Consequently, patients with persistent AF and a larger left atrium may have a higher AF burden and fewer chances of a typical atrial flutter originating from the right atrium. Second, AF and typical atrial flutter commonly occur in combination, with AF leading to atrial flutter and vice versa (13). In other words, typical atrial flutter is frequently preceded by AF, and the intercaval block of the right atrium is linked to the transition from AF to CTI-dependent macro-reentry (5, 13, 14). Typical atrial flutter also easily disorganizes into AF because atrial flutter with a short cycle length may transition to fibrillatory conduction (14). Therefore, CTI ablation may reduce AF recurrence by preventing the organization of AF into atrial flutter, which disrupts AF maintenance (5, 15–17). Finally, linear ablation of the CTI may have affected the right atrial ganglionated plexus. The largest number of ganglia are reported to be located on the posterior surface of the RA, and a previous study of right atrial ganglionated plexi ablation in paroxysmal AF revealed a significant vagal denervation and reduction of AF recurrence (18, 19). Therefore, CTI ablation may also affect vagal denervation in selected patients.

### 4.4. Clinical implications

The main findings of this study can be used to guide clinical practice. In patients with AF, concurrent typical atrial flutter may be a favorable marker for lower AF recurrence, which should be understood for making informed decisions about pursuing rhythm control. That is, patients with AF and concurrent typical atrial flutter commonly feature favorable factors, such as paroxysmal AF with a smaller left atrium, which encourages active rhythm control. In addition, even if a typical atrial flutter is not documented, active induction of concurrent atrial flutter should be performed in patients scheduled for catheter ablation. If typical atrial flutter is induced or detected on a clinical ECG, catheter ablation should be considered to create a linear lesion with a bidirectional CTI block. If the use of additional radiofrequency energy is not feasible, catheter ablation using a cryoenergy source may be an alternative strategy with similar efficacy (6, 20–22).

### 4.5. Limitations

This study had several limitations. First, the proportion of patients with typical atrial flutter was lower than the prevalence of concurrent atrial flutter. Patients who were routinely screened for documented or induced atrial flutter were included in the analysis; however, atrial flutter was detected in only one-fourth of the patients. A major reason for the relatively low prevalence of concurrent atrial flutter may be the increased time and cost of CTI ablation using cryoballoon ablation. Second, this study focused on the presence of typical atrial flutter and did not consider whether CTI ablation should be performed. All patients with typical concurrent atrial fibrillation underwent CTI ablation with acute procedural success (bidirectional CTI block). Therefore, we could not determine the independent effects of concurrent atrial flutter or CTI ablation on the recurrence of atrial arrhythmia. However, previous randomized trials reported no significant benefits of prophylactic CTI ablation in patients undergoing RFCA for AF (12). Therefore, CTI ablation may not yield equivalent benefits in patients without a typical atrial flutter. Third, owing to the retrospective nature of the study, periprocedural and postprocedural management varied among the centers. For example, if typical atrial flutter was clinically diagnosed with a 12-lead ECG, further programmed electrical stimulation was not mandated to perform CTI ablation, which might have included focal or reentry atrial tachycardia other than CTI-dependent tachycardia. In addition, monitoring, or the use of antiarrhythmic drugs during the follow-up period, was not strictly protocolized, which may have underestimated the actual incidence of atrial arrhythmia recurrence. The absence of routine ambulatory monitoring may be biased in favor of patients who present mostly with typical atrial flutter, since atrial flutter is more likely to have a persistent form, and atrial flutter-related symptoms may be greatly reduced after catheter ablation, leading to more lenient rhythm surveillance. Nonetheless, various clinical approaches during and after the procedure reflect the outcomes in real-world practice. Further prospective studies are needed to overcome the biases in the retrospective design and validate our findings. Lastly, this study was limited to patients who underwent cryoballoon ablation, and our results may not be generalizable to other rhythm control strategies, such as antiarrhythmic medication or direct current cardioversion. Nonetheless, the major findings of this study can be applied to patients scheduled for *de novo* catheter ablation for drug-refractory AF, including both cryoballoon and radiofrequency catheter ablations.

## 5. Conclusion

Among patients with drug-refractory AF who underwent cryoballoon ablation, the presence of concurrent typical atrial flutter and additional CTI ablation resulted in a significantly lower recurrence of atrial arrhythmia, mainly driven by a lower recurrence of AF. Patients with typical concurrent atrial flutter share favorable markers for rhythm control, such as a less persistent form of AF and a smaller left atrium. In patients that have coexisting typical atrial flutter, screening for typical atrial flutter should be performed during catheter ablation.

## Data Availability

The original contributions presented in the study are included in the article/**Supplementary Material**; further inquiries can be directed to the corresponding author.
