# Supplementary material for: Association of typical atrial flutter and cavotricuspid isthmus ablation on clinical recurrence after cryoballoon ablation for atrial fibrillation
Source: Front Cardiovasc Med. 2023 Dec 15;10:1303635. doi: 10.3389/fcvm.2023.1303635 (PMC10755020; doi:10.3389/fcvm.2023.1303635)
Supplement: Supplementary file 1 [file Table1.docx]

**SUPPLEMENTARY MATERIALS**

**Supplementary Figure 1. Plot for absolute standardized mean differences.**


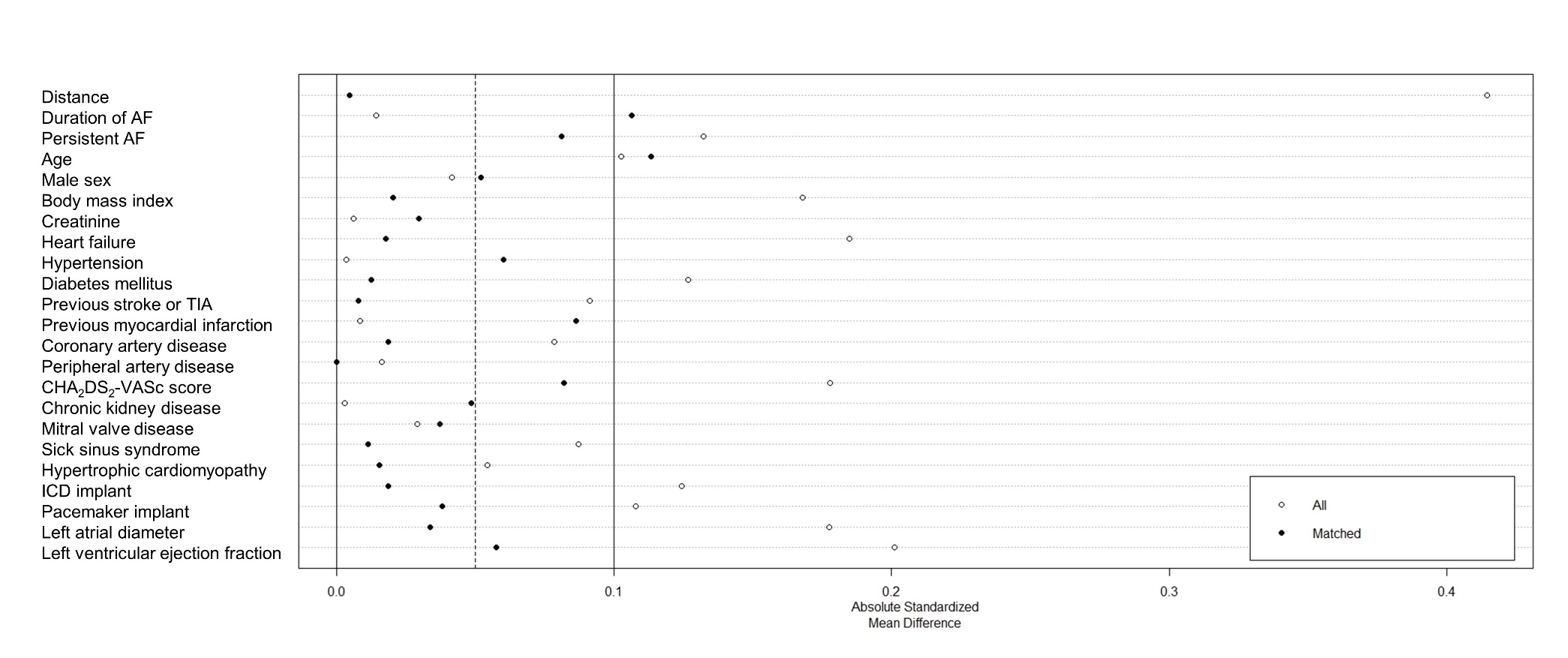


AF, atrial fibrillation; TIA, transient ischemic attack; ICD, implantable cardioverter defibrillator.
